# Predicted risks of radiogenic cardiac toxicity in two pediatric patients undergoing photon or proton radiotherapy

**DOI:** 10.1186/1748-717X-8-184

**Published:** 2013-07-23

**Authors:** Rui Zhang, Rebecca M Howell, Kenneth Homann, Annelise Giebeler, Phillip J Taddei, Anita Mahajan, Wayne D Newhauser

**Affiliations:** 1 The University of Texas Graduate School of Biomedical Sciences at Houston, Houston, TX, USA; 2 Departments of Radiation Physics and Radiation Oncology, Unit 1210, The University of Texas MD Anderson Cancer Center, Houston, TX, USA; 3 Department of Radiation Oncology, Faculty of Medicine, American University of Beirut, Beirut, Lebanon; 4 Medical Physics Program, Department of Physics and Astronomy, Louisiana State University, Baton Rouge, LA, USA; 5 Mary Bird Perkins Cancer Center, Baton Rouge, LA, USA

**Keywords:** Cardiac toxicity, Hodgkin disease, Medulloblastoma, Mediastinal irradiation, Craniospinal irradiation, Proton therapy, Normal tissue complication probability

## Abstract

**Background:**

Hodgkin disease (HD) and medulloblastoma (MB) are common malignancies found in children and young adults, and radiotherapy is part of the standard treatment. It was reported that these patients who received radiation therapy have an increased risk of cardiovascular late effects. We compared the predicted risk of developing radiogenic cardiac toxicity after photon versus proton radiotherapies for a pediatric patient with HD and a pediatric patient with MB.

**Methods:**

In the treatment plans, each patient’s heart was contoured in fine detail, including substructures of the pericardium and myocardium. Risk calculations took into account both therapeutic and stray radiation doses. We calculated the relative risk (*RR*) of cardiac toxicity using a linear risk model and the normal tissue complication probability (*NTCP*) values using relative seriality and Lyman models. Uncertainty analyses were also performed.

**Results:**

The *RR* values of cardiac toxicity for the HD patient were 7.27 (proton) and 8.37 (photon), respectively; the *RR* values for the MB patient were 1.28 (proton) and 8.39 (photon), respectively. The predicted *NTCP* values for the HD patient were 2.17% (proton) and 2.67% (photon) for the myocardium, and were 2.11% (proton) and 1.92% (photon) for the whole heart. The predicted ratios of NTCP values (proton/photon) for the MB patient were much less than unity. Uncertainty analyses revealed that the predicted ratio of risk between proton and photon therapies was sensitive to uncertainties in the *NTCP* model parameters and the mean radiation weighting factor for neutrons, but was not sensitive to heart structure contours. The qualitative findings of the study were not sensitive to uncertainties in these factors.

**Conclusions:**

We conclude that proton and photon radiotherapies confer similar predicted risks of cardiac toxicity for the HD patient in this study, and that proton therapy reduced the predicted risk for the MB patient in this study.

## Background

Hodgkin disease (HD) and medulloblastoma (MB) are among the most common malignancies found in children and young adults, and radiotherapy (mediastinal radiation for HD and radiotherapy to the cranium and spine for MB) is part of the standard treatment. Coronary vascular disease was found to be associated with a higher radiation dose in HD survivors [1], and the leading cause of noncancer mortality in radiation-treated HD patients is cardiovascular disease [2,3]. It was found that craniospinal irradiation (CSI) patients are at risk for significant cardiac dysfunction and asymmetric impairment of heart development, where asymmetric distribution of radiation may be the cause [4]. Pediatric survivors who received radiotherapy and chemotherapy for brain tumors are at increased risk for cardiovascular late effects [5].

Proton therapy typically delivers a lower dose to normal tissues than photon therapy [6]. However, stray radiation dose from neutrons generated during proton therapy is of concern [7,8]. Realistic calculations of stray radiation dose within a patient are computationally complex and have only recently been accomplished for proton therapy [8-10]. Recently, there has been much progress in research to compare the risks of second cancers after photon and proton radiotherapies [8,10-13]. In contrast, relatively little attention has been paid in the literature to predictive comparisons of other late effects, such as cardiac toxicity [11], for pediatric patients who received radiotherapy.

The aim of this work was to compare the predicted risks of cardiac toxicity after photon versus proton radiotherapies for a pediatric HD patient and a pediatric MB patient. Both therapeutic and secondary radiation doses were included in the risk predictions. We calculated the therapeutic photon and proton absorbed doses and secondary photon absorbed doses using a commercial treatment planning system (TPS); the secondary radiation dose for proton therapy was obtained from Monte Carlo simulations. Dose-risk models from the literature were used to estimate the risk of cardiac toxicity, and rigorous uncertainty analyses were carried out on risk predictions.

## Methods

### Study patients and treatment techniques

For the purposes of this study, we created new proton and photon treatment plans using the same commercial TPS (Eclipse version 8.9, Varian Medical Systems, Palo Alto, CA) for two patients who were formerly treated at The University of Texas MD Anderson Cancer Center following protocol approved by our institutional review board. Both patients were treated with passively scattered proton therapy with treatment plans having the same beam arrangements as those planned for this study, but were originally planned using an earlier version of the TPS software. Our proton therapy facility uses a synchrotron accelerator and passive scattered proton beams (PSPT) were used for the treatment plans in this study. The photon treatment plans in this study were planned for 6MV beams delivered using a linear accelerator (Varian 2100, Palo Alto, CA) equipped with a 120 leaf multileaf collimator (Millenium, Varian, Palo Alto) [14,15]. We decided not consider intensity modulated proton therapy (IMPT) because at the time the study was conducted, IMPT was not in clinical use for HD or MB. Therefore, this would have reduced the clinical relevance of the paper. Furthermore, the management of organ motion and interplay effects with scanned beams are not well understood or sufficiently developed to allow a comparison of comparable levels of radiotherapy technologies. Furthermore, IMPT is not even available at many proton centers.

The first patient was a 10-year-old girl diagnosed with HD. The clinical target volume (CTV) was delineated by the radiation oncologist and included demonstrated tumor and tissue with presumed tumor [16]. The proton treatment plan comprised parallel opposed anterior-posterior (AP) and posterior-anterior (PA) fields having energies of 140 and 180 MeV, respectively. A range compensator and collimating aperture for each field were separately designed to achieve coverage of the CTV while including allowances for uncertainties in beam range, penumbra, patient set-up, and potential target motion. The photon plan included five co-planar intensity-modulated beams with five gantry angles (0°, 20°, 170°, 190°, and 340°), selected to minimize dose in the lungs. The CTV was expanded 5 mm isotropically to create a planning target volume (PTV) for the photon plan, and the plan was optimized using the inverse planning method. The total prescribed dose (according to National Cancer Center Network (NCCN) guideline) was 36 Gy relative biological effectiveness (RBE) (i.e., 32.7 Gy × 1.1 to reflect the biological effectiveness of protons relative to photons) and 36 Gy for the proton and photon treatment plans, respectively; 18 fractions were used for both treatment plans.

The second patient was a 4-year-old boy who was diagnosed with MB. His proton treatment plan included right and left posterior oblique cranial fields (gantry angles 105° and 255°) and two PA spinal fields. The target volume included the cranial and spinal cavities and entire vertebral body (to prevent asymmetric bone growth caused by non-uniform dose distribution in the vertebral bodies). The photon treatment plan consisted of two opposed lateral cranial fields (gantry angles 90° and 270°) and one PA spinal field. The beam energy for all photon fields was 6 MV, and the plan included junction shifts after 9 Gy and 16.2 Gy. The prescribed dose was 23.4 Gy (RBE) and 23.4 Gy for the proton and photon treatment plans, respectively; 13 fractions were used for both treatment plans. Additional details of the CSI treatment planning can be found elsewhere [14,15].

The external surface of the heart was contoured in every computed tomography (CT) slice from the inferior border of the right pulmonary artery to the apex of the heart. The pericardium was defined as a 2-mm shell inside the external heart surface contours. The myocardium was a shell having an external contour identical to the internal contour of the pericardium and a thickness varying from 1 cm to 2 cm, with the thickness on the left side being twice that on the right side.

### Dose reconstruction and risk calculation

The therapeutic doses from photon and proton plans were estimated from the TPS directly. The secondary dose from proton therapy was calculated by our Monte Carlo Proton Radiotherapy Treatment Planning system, which uses the Monte Carlo N-particle eXtended code (MCNPX, version 2.6, Los Alamos National Laboratory) [17] as a dose engine. Details of this system were previously described [8]. The secondary dose from the photon plan was obtained from the TPS for the following reasons. Howell et al. [18] recently reported that the TPS used in this work (for 6 MV) was accurate to the level of 5% of the prescribed dose. In this study, the heart and major cardiac substructures were entirely within the 5% isodose line for both patients; consequently, it was feasible to accurately assess the organ dose directly using the TPS. The equivalent dose in each organ, *H*_T_, was calculated by multiplying the organ dose, *D*_*T*_, by a mean radiation weighting factor, $\overset{—}{w_{R}}$. The $\overset{—}{w_{R}}$ values were taken as 1.1 for proton primary fields-that is, dose values were reported in Gy (RBE)-and 1.0 for photon fields. The mean radiation weighting factor $\overset{—}{w_{R}}$ values for neutrons were obtained from Monte Carlo simulations (unpublished data) and previous report [8], following the methods in literature [8,19].

Based on modeling methods we found in a comprehensive search of the literature, we calculated the relative risks (*RR*) of radiogenic cardiac toxicity using the linear model [20] (*α*_1_ = 0.6, 95% confidence interval [CI], 0.2 to 2.5) and normal tissue complication probability (*NTCP*) using relative seriality (RS) [21] and Lyman [22] models. (The models are summarized in the Appendix.)

Table 1 lists the *NTCP* model parameter sets from the literature. The model parameters are from patients treated for HD [23], breast cancer [24], esophageal cancer [25], and historical outcome data [26]. Only the parameters based on HD data were used in the risk calculations for the HD patient in this study. However, to our knowledge, the literature contains no report that establishes a detailed relationship between radiation dose and the incidence of cardiac toxicity for MB patients. Consequently, *NTCP* model parameters specific to MB patients were not available. Thus, all the possible sets of parameters in Table 1 were tried for the MB patient, but the ratios of *NTCP* (*RNTCP*), from proton therapy to photon therapy, were presented instead of *NTCP* values themselves, because of the large uncertainties associated with the *NTCP* values.

**Table 1:** **Predicted *NTCP *and *RNTCP *values for cardiac structures based on existing model parameters; for the HD patient, *NTCP *and *RNTCP *values based on correct literature parameters were listed; for the MB patient, calculations were carried out by using all possible parameter sets, and only *RNTCP *values were listed**

							**HD patient**			**MB patient**
**Structure**	**Ref.**	***D***_**50 **_**(Gy)**	***n***	***m***	**γ**	***s***	***NTCP***_**Proton **_**(%)**	***NTCP***_**Photon **_**(%)**	***RNTCP***	***RNTCP***
Pericardium	[26]	48	0.35	0.1	-	-	-	-	-	0
Pericardium	[25]	50.6	0.64	0.13	-	-	-	-	-	1.43 × 10^-5^
Myocardium	[24]	52.2	-	-	1.25	0.87	-	-	-	1.93 × 10^-5^
Myocardium	[23]	70.3	-	-	0.96	1	2.17	2.67	0.8	0.03
Whole heart	[24]	52.3	-	-	1.28	1	-	-	-	1.66 × 10^-4^
Whole heart	[23]	63.3	-	-	0.93	1	-	-	-	0.032
Whole heart	[23]	70.3	-	-	0.96	1	2.11	1.92	1.1	0.028

To compare risk values between photon and proton plans, we defined the ratio of *RR* (*RRR*), based on the linear model, as

(1) $$\text{RRR}=RR_{\text{proton}}/RR_{\text{photon}},$$

and the *RNTCP* was defined as

(2) $$\text{RNTCP}=\text{NTC}P_{\text{proton}}/\text{NTC}P_{\text{proton}}.$$

The mean whole-heart doses were used to calculate *RR* values. Dose volume histograms (DVHs) for heart structures were exported from the TPS to calculate the corresponding *NTCP* values. For the photon plan, the DVHs were taken from the TPS directly. For the proton plan, the DVHs for primary dose were obtained from the TPS and a mean neutron equivalent dose (neutron absorbed dose multiplied by neutron $\overset{—}{w_{R}}$) was then added uniformly to the primary DVH, which was appropriate considering the secondary neutron dose was spatially nearly uniform. Each step in the differential DVH was corrected to a 2 Gy or Gy (RBE)/fraction schedule by using the linear quadratic model [27]. An *α/β* ratio of 3 was chosen for the late effects in the heart [23].

### Uncertainty analysis

There are potentially large and poorly known uncertainties in the *NTCP* model parameters for the MB patient. We performed a sensitivity test of the predicted *RNTCP* values to these uncertainties by varying each model parameter over its plausible range (see Table 2).

**Table 2:** **Predicted *RNTCP *values for cardiac structures for the MB patient using combinations of *NTCP *model parameters (min: *D***_**50**_ **= 40 Gy, *n*** **= 1, *m*** **= 0.05; max: *D***_**50**_ **= 80 Gy, *n*** **= 0.2, *m*** **= 0.5), the myocardium (min: *D***_**50**_ **= 40 Gy, *γ*** **= 1.5, *s*** **= 0.5; max: *D***_**50**_ **= 80 Gy, *γ*** **= 0.8, *s*** **= 1), and the whole heart (min: *D***_**50**_ **= 40 Gy, *γ*** **= 1.5, *s*** **= 0.5; max: *D***_**50**_ **= 80 Gy, *γ*** **= 0.8, *s*** **= 1)**

**Structure**	***RNTCP***		
	**Minimum**	**Baseline**	**Maximum**
Pericardium	0	1.43 × 10^-5^	0.57
Myocardium	0	0.03	0.19
Whole heart	0	0.03	0.18

**Figure 1:**
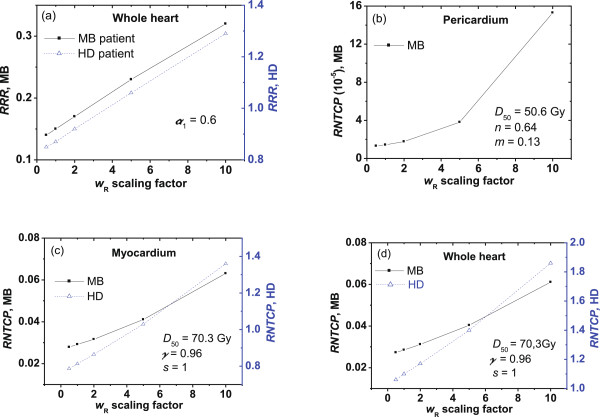
**Sensitivity tests.** Sensitivity of the predicted **(a)***RRR* values, and *RNTCP* values for the **(b)** pericardium, **(c)** myocardium, and **(d)** whole heart to changes in the neutron radiation weighting factor $\overset{—}{w_{R}}$. The *NTCP* model parameters used for the calculations are listed in each panel.

There are large uncertainties associated with neutron $\overset{—}{w_{R}}$. The International Commission on Radiological Protection [28] recommended in Publication 92 that the maximum neutron *w*_*R*_ should be 20. We performed a sensitivity test to quantify the impact of uncertainty on the $\overset{—}{w_{R}}$ values by using different scaling factors in cardiac toxicity calculations (see Figure 1).

**Figure 2:**
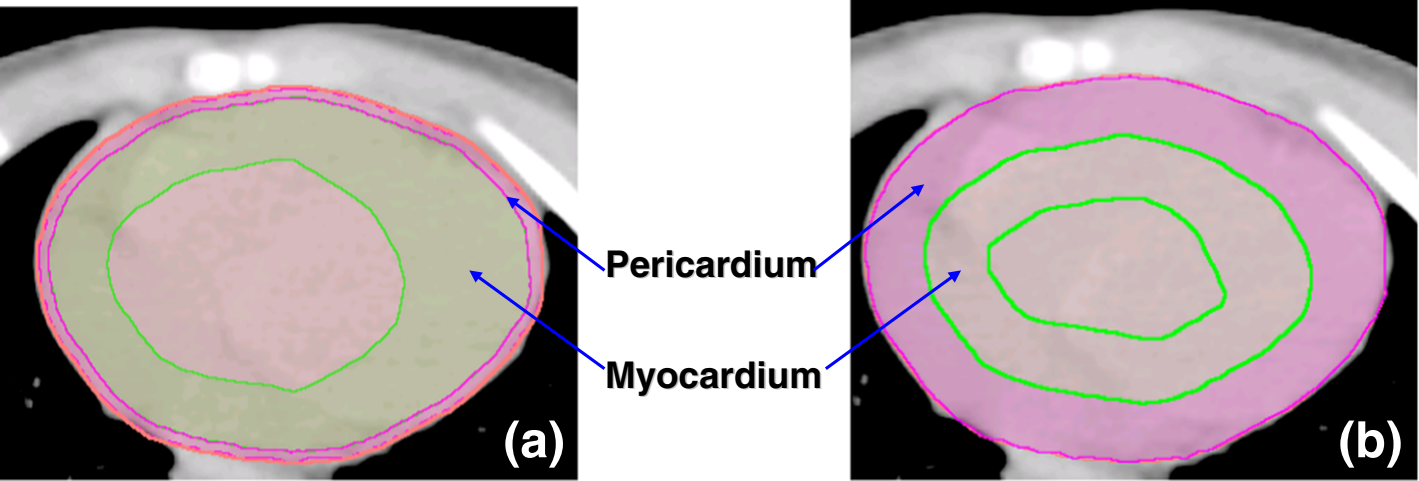
**Heart substructure contouring: (a)** baseline contouring, and **(b)** revised contouring that was used for uncertainty analysis.

Contouring of heart substructures was challenging and may have introduced large uncertainties. In the prevailing standard of care, CT imaging methods used in treatment planning for external beam radiotherapy do not clearly show these substructures. The methods for radiographic identification and delineation of the heart substructures are not standardized, and knowledge of the uncertainty in the contouring process is incomplete [24,25,29]. For these reasons, we performed sensitivity tests to quantify the influence of heart contouring on the *RNTCP* values. We redefined the pericardium as a 1-cm shell (*i.e.*, 5 times thicker than the shell in the baseline calculations) inside the external heart surface and the myocardium to be a 1-cm shell inside the inner surface of the thicker pericardium (Figure 2).

## Results

**Figure 3:**
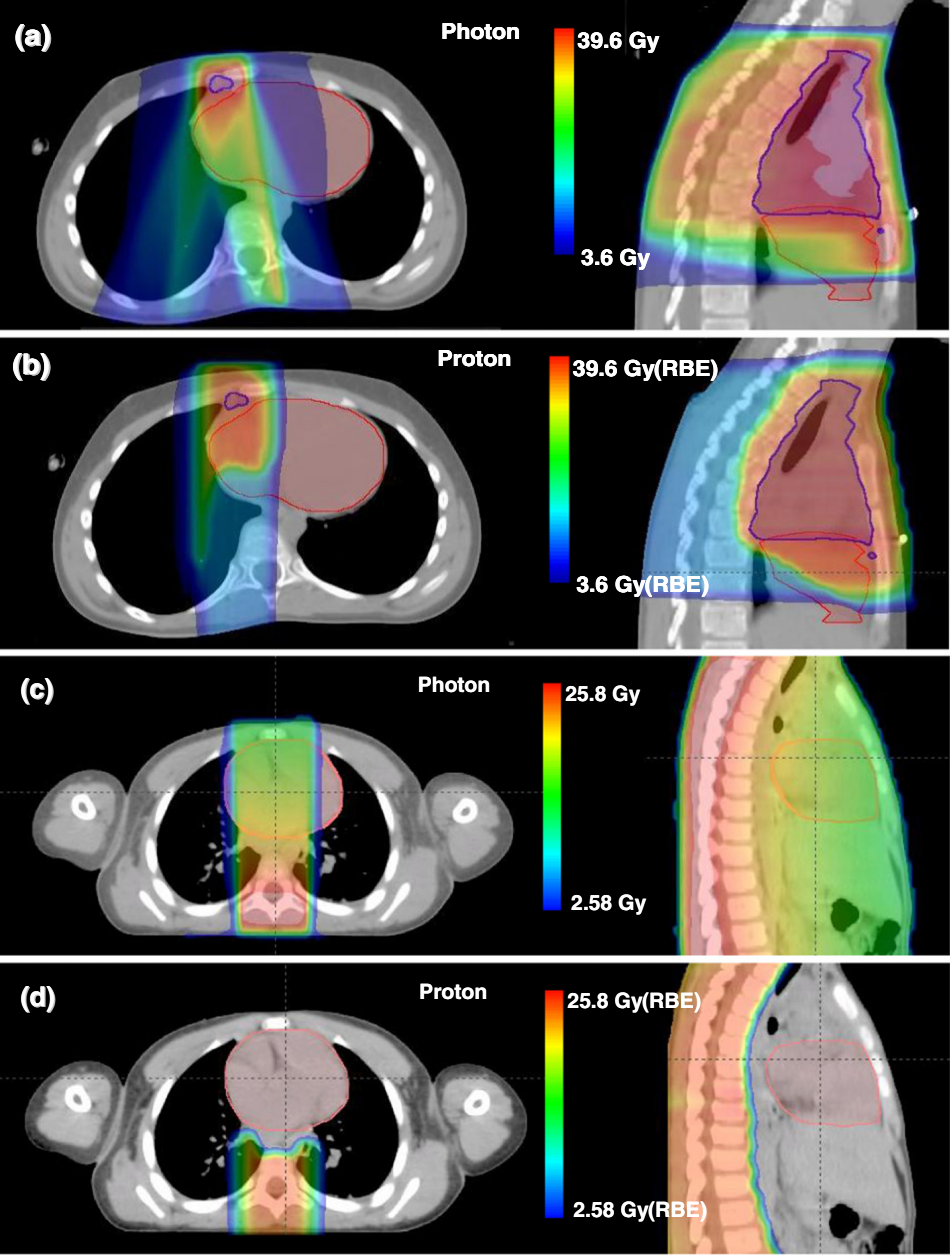
**Axial and sagittal slices of dose distributions from the photon and proton plans. (a**, **b)** Slices for the HD patient and **(c**, **d)** slices for the MB patient. Heart contour (red) was displayed for both patients, and CTV contour (blue) was displayed for the HD patient.

**Figure 4:**
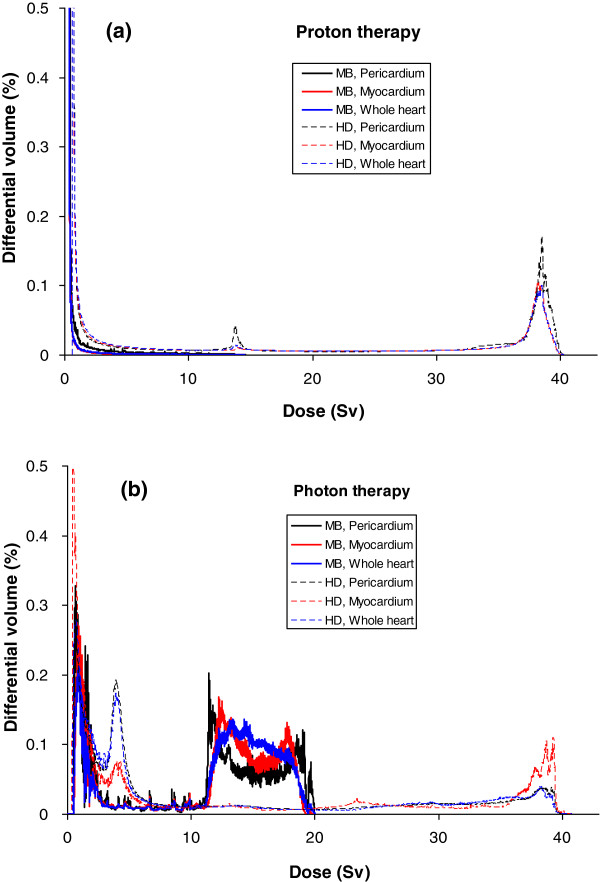
**Differential DVHs of the whole heart and heart substructures.** DVHs from **(a)** proton and **(b)** photon treatment plans for the MB patient and the HD patient. For the photon plan, the DVHs were taken from TPS directly; for the proton plan, the DVHs for primary dose were obtained from TPS, then a mean neutron equivalent dose was added uniformly to the primary DVHs.

Figure 3 shows therapeutic dose distributions in the heart area from photon and proton treatment plans, clearly revealing that proton beams provided a much lower exit dose to the heart for the MB patient, while comparable doses were delivered to the heart from both plans for the HD patient. The mean organ doses to the heart substructures are listed in Table 3, including the secondary neutron doses from proton therapy. Figure 4 shows the differential DVHs from photon and proton plans.

**Table 3:** Mean organ doses (Gy) and equivalent doses (Sv) to heart and substructures from proton and photon plans for the pediatric MB patient and HD patient

**Patient**	**Structure**	**Proton therapy dose (Gy)**		**Proton therapy dose (Sv)**			**Photon therapy dose (Gy)**	**Photon therapy dose (Sv)**
		**Primary**	**Stray**	**Primary**	**Stray**	**Total**		
	Pericardium	0.55	0.032	0.61	0.26	0.87	10.59	10.59
MB	Myocardium	0.13	0.033	0.14	0.26	0.40	11.72	11.72
	Whole heart	0.19	0.032	0.21	0.26	0.47	12.31	12.31
	Pericardium	11.15	0.081	12.27	0.69	12.96	14.26	14.26
HD	Myocardium	9.05	0.077	9.95	0.65	10.60	12.23	12.23
	Whole heart	8.90	0.078	9.79	0.66	10.45	12.28	12.28

The *RR* values of cardiac toxicity following proton and photon therapies for the HD patient were 7.27 (95% CI, 3.09 to 27.12) and 8.37 (95% CI, 3.46 to 31.70), respectively, and the corresponding *RRR* was 0.87. The *RR* values following proton and photon CSI for the MB patient were 1.28 (95% CI, 1.09 to 2.18) and 8.39 (95% CI, 3.46 to 31.78), respectively, and the corresponding *RRR* was 0.15.

Table 1 lists the predicted *NTCP* and *RNTCP* values for the heart structures. The *NTCP* values for the HD patient were 2.17% (proton) and 2.67% (photon) with the myocardium as the organ at risk, and were 2.11% (proton) and 1.92% (photon) with the whole heart as the organ at risk. The predicted *RNTCP* values for the MB patient were always much less than unity, regardless of the parameter sets used.

**Figure 5:**
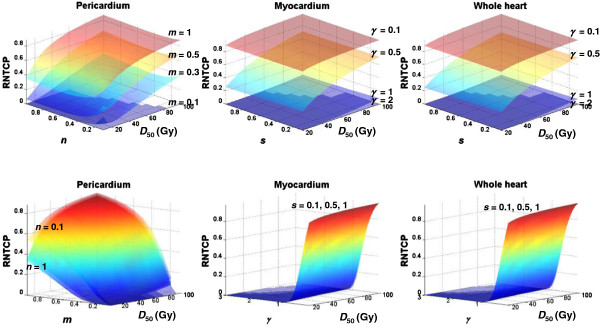
**Surfaces of predicted *RNTCP *values for heart substructures as functions of different *NTCP *parameters for the MB patient.** (**Top**) The surfaces displayed were calculated for *m* values of 0.1, 0.3, 0.5, and 1 (pericardium) and *γ* values of 0.1, 0.5, 1, and 2 (myocardium and whole heart). (**Bottom**) The surfaces displayed were calculated for *n* values of 0.1, 0.3, 0.5, and 1 (pericardium) and *s* values of 0.1, 0.5, and 1 (myocardium and whole heart). Color has been interpolated to facilitate visualization.

Figure 5 plots *RNTCP* values for the whole heart, myocardium, and pericardium for the MB patient, using various *NTCP* model parameters. The Lyman model was tested for the pericardium, while the RS model was tested for the myocardium and the whole heart. For the pericardium, the *RNTCP* values were not sensitive to changes in *n* values but were sensitive to changes in *D*_50_ values and very sensitive to changes in *m* values (as *m* increased from 0.1 to 1, the *RNTCP* increased substantially). For the myocardium and the whole heart, the *RNTCP* values were not sensitive to changes in *D*_50_ or *s* values but were very sensitive to changes in *γ* values (as *γ* increased from 0.1 to 2, the *RNTCP* decreased substantially). Given these results, we selected reasonable combinations to estimate the minimum and maximum *RNTCP* values (Table 2). The intervals for the *NTCP* model parameters were set large enough to include all published parameters from the literature. Results of the *RNTCP* calculations are listed in Table 2, and they are all less than unity.

The sensitivity of *RRR* to scaling factor of neutron $\overset{—}{w_{R}}$ values and the sensitivity of *RNTCP* to the $\overset{—}{w_{R}}$ scaling factor are shown in Figure 1. The *RRR* value changed between 0.85 and 1.29 for the HD patient as the $\overset{—}{w_{R}}$ value was scaled from 0.5 times to 10 times the nominal value; they were less than unity for the MB patient in all cases considered. The *RNTCP* values varied between 0.79 and 1.36 for the HD patient with myocardium as the organ at risk, and they varied between 1.06 and 1.86 for the HD patient with the whole heart as the organ at risk. The *RNTCP* values were all much less than unity for the MB patient.

The *RNTCP* calculations obtained when using different heart-contouring methods are listed in Table 4. In all cases, the predicted *RNTCP* values did not change much from the baseline for both patients.

**Table 4:** **Predicted *RNTCP *values for cardiac structures for the HD patient and the MB patient when using revised contours**

**Structure**	**HD patient**		**MB patient**	
	***RNTCP***	***RNTCP***	***RNTCP***	***RNTCP***
	**(baseline)**	**(revised)**	**(baseline)**	**(revised)**
Pericardium	-	-	1.43 × 10^-5^	1.49 × 10^-5^
Myocardium	0.8	1.27	0.03	0.024
	(*NTCP*_Proton_ = 2.17%, *NTCP*_Photon_ = 2.67%)	(*NTCP*_Proton_ = 1.24%, *NTCP*_Photon_ = 0.98%)		

## Discussion

We predicted risks of cardiac toxicities for a 4-year-old boy receiving photon or proton CSI for MB and a 10-year-old girl receiving photon or proton therapy for HD. Therapeutic and stray radiation doses were considered. To our knowledge, this study incorporated, for the first time, patient-specific stray neutron doses into the *NTCP* calculations. We used published model parameters in the risk calculations, and we examined the sensitivity of *RR* values to *NTCP* model parameter values, the neutron $\overset{—}{w_{R}}$ values and heart contouring. The major finding of this study is that proton and photon therapies conferred comparable predicted risk of radiogenic cardiac toxicity for the HD patient, and proton therapy reduced the risk for the MB patient compared to photon therapy.

Our predicted *RR* and *NTCP* values for the HD patient agree well with those in the literature, in which the relative risks of cardiac mortality among HD patients range from 2.2 to 12.7 [3], and the relative risk of myocardial infarction of pediatric HD survivors is reported as 12.2 (95% CI, 5.2 to 28.2) [30]. Those data agree well with our calculated *RR* of 8.37 (95% CI, 3.46 to 31.7) for the photon plan, considering that most of the historical treatments used photon techniques. The absolute excess risk of cardiac mortality ranges from 0.093% to 0.5% per year among HD patients in the literature [3,31], which means a 0.47% to 2.5% risk in the first 5 years after exposure. Our predicted *NTCP* values at 5 years after exposure were 2.67% for myocardium and 1.92% for the whole heart. Again, they are in reasonable agreement with the literature.

The *RRR* and *RNTCP* values for cardiac toxicity were sensitive to the uncertainties in $\overset{—}{w_{R}}$ for neutrons. Especially for the HD patient, the *RRR* and *RNTCP* values were higher than unity when $\overset{—}{w_{R}}$ was scaled to high values, indicating that the predicted risk of cardiac toxicity from proton therapy may be higher than that from photon therapy if the RBE of stray neutrons is large. While the advantage of proton therapy is not obvious in terms of reducing cardiac toxicity for this HD patient, the lower out-of-field dose would possibly decrease risks of other late effects [32,33]. It is possible that risk from proton therapy could be reduced if IMPT were used rather than PSPT. However, we focused on PSPT because it is the technique that is most widely available.

Considering the breadth of *NTCP* model parameters we tested, and the substantial differences in the dose distributions between proton and photon CSI, we interpret the results of this work as suggesting that proton CSI carries a lower risk of cardiac toxicity than photon CSI. In this type of predictive analysis, the calculated *NTCP* values are highly dependent on the clinical data and thus are more suitable for comparing the relative risks of treatments rather than predicting absolute outcomes [34].

This study has some limitations. First, we used a single case for each type of disease. While CSI is the standard for MB treatment, various treatment fields exist for HD patients. However, the emphasis of this paper is on the methodology and this paper is the preliminary report of a follow-up cohort study, which is being carried out by our group. Second, we applied risk models that carry large uncertainties to the MB patient. However, our sensitivity test revealed the robustness of the qualitative finding that proton CSI carries a lower risk of cardiac toxicity than photon CSI. Third, the heart contouring was simplified in this study, but we believe this kind of simplification is warranted, given the large uncertainties associated with the *NTCP* model itself. Again, our sensitivity tests of the effects of varying the heart contouring strengthened our conclusion. Finally, we only considered radiation induced cardiac toxicity because this is the focus of this study, while chemotherapy was also reported to significantly increase risk of cardiac toxicity [20,35]. However, according to Tukenova et al. [20], the radiation dose did not significantly interact with chemotherapy dose, which justified the risk calculations in our study.

## Conclusion

Proton therapy conferred a similar risk of radiogenic cardiac toxicity as photon therapy for the HD patient in this study, while substantially reducing the risk for the MB patient in this study. Sensitivity analyses revealed that *RRR* and *RNTCP* values were sensitive to uncertainty in the mean neutron $\overset{—}{w_{R}}$ values, and *RNTCP* was sensitive to the *NTCP* model parameters but not sensitive to variations in the heart structure contours. The qualitative findings of the study were not sensitive to the uncertainties in these factors.

## Appendix

Tukenova et al. [20] reported a relationship between the mean radiation dose to the heart and the risk of cardiac mortality based on a large sample of follow-up data on childhood cancer survivors and they concluded the best dose–response model was:

(3) $$\text{RR}=1+\alpha_{1}D,$$

where *RR* is the relative risk, *D* is the mean heart dose, and *α*_1_, which is the linear coefficient, is 0.6 (95% CI, 0.2 to 2.5). The same group reported a linear model to estimate risk of cardiac disorder and a linear quadratic model to estimate risk of cardiac failure in 2006 [36]. Considering that reference [20] is the most up-to-date, the linear model in it was chosen for this study.

The relative seriality model is based on the Poisson model of cell survival [21]. The probability of cell death when irradiating a tissue to dose *D* is

(4) $$P\left(D\right)=2^{-\exp\left\{\mathrm{e\gamma}\left(1-D/D_{50}\right)\right\}},$$

where *γ* is the maximum relative slope of the dose–response curve and *D*_50_ is the dose that will result in a 50% probability of a complication. The normal tissue complication probability (*NTCP*) due to inhomogeneous irradiation is given by

(5) $$\text{NTCP}={\left\{1-\prod_{i=1}^{n}{\left[1-P{\left(D_{i}\right)}^{s}\right]}^{V_{i}/V}\right\}}^{1/s},$$

where *s* is the relative seriality that describes the hybrid serial/parallel architecture of the organ, (*s* = 0 indicates parallel organization, while *s* = 1 indicates serial organization), *n* is the number of voxels in the dose-calculation volume, *D*_*i*_ is the dose in each subvolume, *V*_*i*_ is the volume of each subvolume in the differential dose-volume histogram, and *V* is the total volume of the organ.

The Lyman model [22] assumes that the probability of complication is a normal distribution as a function of dose to the partially irradiated volume *V*. The *NTCP* is given by

(6) $$\text{NTCP}=\frac{1}{\sqrt{2\pi }}\int_{-\infty }^{t}e^{-t^{2}/2dt}$$

(7) $$t=\left(D-TD_{50}\left(V\right)\right)/\left(m\cdot TD_{50}\left(V\right)\right)$$

(8) $$TD_{50}\left(V\right)=TD_{50}\left(1\right)/V^{n}$$

where *TD*_50_(*V*) is the tolerance dose that would result in a 50% complication probability for the partial volume *V*, *TD*_50_(1) is the tolerance dose that would result in a 50% complication probability for the full organ, *n* indicates the volume effect (*n* close to 1 means the volume effect is strong), and *m* is inversely proportional to the slope of the dose–response curve.

## Competing interests

The authors declare that they have no competing interests.

## Authors’ contributions

RZ participated in the design of the project, participated in dose reconstructions, did the risk calculations and data analysis, and drafted/edited the manuscript. RH participated in creating photon plans and assisted with reviewing/editing the manuscript. KH participated in creating proton and photon plans and dose reconstructions. AG participated in creating proton plans. PT assisted with reviewing/editing the manuscript and participated in dose reconstructions. AM provided guidance on treatment plan design and approved all treatment plans. WN designed the project, assisted in reviewing/editing the manuscript and provided funding. All authors read and approved the final manuscript.
